# Association between Body Mass Index and Outcomes in Patients with Return of Spontaneous Circulation after Out-of-Hospital Cardiac Arrest: A Systematic Review and Meta-Analysis

**DOI:** 10.3390/ijerph18168389

**Published:** 2021-08-08

**Authors:** Heekyung Lee, Hyungoo Shin, Jaehoon Oh, Tae-Ho Lim, Bo-Seung Kang, Hyunggoo Kang, Hyuk-Joong Choi, Changsun Kim, Jung-Hwan Park

**Affiliations:** 1 Department of Emergency Medicine, College of Medicine, Hanyang University, Seoul 04763, Korea; massdt@hanyang.ac.kr (H.L.); newnowold@hanyang.ac.kr (H.S.); erthim@hanyang.ac.kr (T.-H.L.); olivertw@hanyang.ac.kr (B.-S.K.); emer0905@hanyang.ac.kr (H.K.); ardoc@hanyang.ac.kr (H.-J.C.); flyes98@hanyang.ac.kr (C.K.); 2 Department of Internal Medicine, College of Medicine, Hanyang University, Seoul 04763, Korea; parkjh0616@hanyang.ac.kr

**Keywords:** body mass index, obesity, heart arrest, cardiopulmonary resuscitation, patient outcome assessment, meta-analysis

## Abstract

Increased body mass index (BMI) is a risk factor for cardiovascular disease, stroke, and metabolic diseases. A high BMI may affect outcomes of post-cardiac arrest patients, but the association remains debatable. We aimed to determine the association between BMI and outcomes in patients with return of spontaneous circulation (ROSC) after out-of-hospital cardiac arrest (OHCA). A systematic literature search was conducted using MEDLINE, EMBASE, and the Cochrane Library. Studies that included patients who presented ROSC after OHCA, had a recorded BMI, and were assessed for neurological outcomes and in-hospital mortality were included. To assess the risk of bias of each included study, we employed the Risk of Bias Assessment Tool for Non-randomized Studies. We assessed 2427 patients from six studies. Neurological outcomes were significantly poorer in underweight patients (risk ratio (RR) = 1.21; 95% confidence interval (CI) = 1.07–1.37; p = 0.002; I^2^ = 51%) than in normal-weight patients. Additionally, in-hospital mortality rate was significantly higher in underweight patients (RR = 1.35; 95% CI = 1.14–1.60; p<0.001; I^2^ = 21%) and in obese patients (RR = 1.25; 95% CI = 1.12–1.39; p<0.001; I^2^ = 0%) than in normal-weight patients. Poor neurological outcome is associated with underweight, and low survival rate is associated with underweight and obesity in patients with ROSC after OHCA.

## 1. Introduction

Over 1.9 billion adults worldwide were estimated to be overweight in 2016, and 31.4% of U.S. adults had obesity in 2019, which is a major global health problem [1,2]. Increased body mass index (BMI) is a risk factor for serious health conditions, including cardiovascular disease, stroke, and metabolic diseases, such as type 2 diabetes mellitus [3,4]. 

High BMI may affect the outcomes of post-cardiac arrest patients. Oxygen consumption, carbon dioxide production, and the risk of rapid desaturation are increased in obese patients [5]. Surgical airway and endotracheal intubation as well as bag-mask ventilation can be difficult in obese patients [6]. Proper hand position and compression depth for high-quality chest compressions may be changed in obese cardiac arrest patients [7]. All of these factors may influence the outcomes of obese cardiac arrest patients compared with those in patients with a normal BMI.

However, previous evidence has shown a U- or J-shaped relationship between better outcomes and BMI, termed the “obesity paradox” [8,9]. There is still a debate on the association between BMI and the outcomes in cardiac arrest patients [8,9]. Additionally, in the underweight population, increased risk of poor prognosis after cardiovascular disease may be associated with various clinical factors, such as aging, sarcopenia, and poor nutritional status [10]. Several previous studies have reported that a low BMI (underweight patient) is associated with poor outcomes in critically ill and post-cardiac-arrest patients [11,12,13].

Recent meta-analyses have shown an impact of BMI on the outcomes of post-cardiac-arrest patients, including both in-hospital (IHCA) and out-of-hospital cardiac arrests (OHCA) [14,15]. However, IHCA and OHCA patients differ in age, comorbidities, initial shockable rhythm, and time to resuscitation [16]. Based on these differences, IHCA patients have better outcomes than OHCA patients [16]. Ma et al. showed no difference in survival conditions with OHCA in the subgroup analysis between overweight or obese patients and normal-weight patients [15]. However, previous meta-analysis studies have various errors in data collection and meta-analysis procedures; for example, in one study, both IHCA and OHCA patients were recruited for subgroup analysis, the included population had different inclusion criteria, and the association between underweight status and outcomes in OHCA patients was not presented [15]. Moreover, several studies comparing the outcomes between BMI categories in OHCA patients have been reported recently [17,18].

Early prediction of outcomes in OHCA patients may help to decide appropriate management plan, such as targeted temperature management (TTM). To the best of our knowledge, a meta-analysis identifying the association between BMI and outcomes in patients with ROSC after OHCA has not been performed yet. Therefore, we focused on and compared the neurological and survival outcomes of OHCA in underweight, normal weight, overweight, and obese patients using a meta-analytical approach.

## 2. Materials and Methods

### 2.1. Protocol and Registration

We followed the Meta-analysis of Observational Studies in Epidemiology and the Preferred Reporting Items for Systematic Reviews and Meta-analysis guidelines [19,20]. The protocol is registered at http://www.crd.york.ac.uk/PROSPERO/ (CRD42021220047).

### 2.2. Eligibility Criteria

This study applied the population, intervention, comparison, and outcome (PICO) search strategy. Using the PICO question, population (P) = in patients with ROSC after OHCA; intervention (I) = BMI; comparator (C) = none; outcome (O) = neurological outcome and in-hospital mortality.

### 2.3. Information Sources and Literature Search Strategy

An extensive database search was performed for studies identifying an association between BMI and outcomes in patients with ROSC after OHCA. Two reviewers (H.L. and H.S.) carried out the literature search on 20 July 2021. The search was conducted using MEDLINE (1946 to 16 July 2021) and Embase (1974 to 16 July 2021) databases via the Ovid interface in addition to the Cochrane databases (all years). We also manually checked the references of all eligible studies to identify other relevant studies. The search terms used were as follows: “cardiac arrest,” “cardiopulmonary resuscitation,” “heart arrest,” “return of spontaneous resuscitation,” “advanced cardiac life support,” and “body mass index,” “obesity,” “overweight,” or “underweight” (Supplementary Table S1). No language restrictions and methodology filters were used.

### 2.4. Study Selection

The title, abstract, and study type of each study were examined by two reviewers (H.L. and H.S.). The exclusion criteria were as follows: irrelevant population (IHCA patients, patients without sustained ROSC), irrelevant outcome measure, irrelevant control group, reviews, case reports, editorials, conference abstracts, meta-analyses, reports with animal studies, or studies on pediatrics. Duplicate articles were excluded manually after comparing titles, authors, and year of publication of all identified studies. In case of a disagreement between the two reviewers, a third reviewer (J.O.) intervened, and differences were discussed until a consensus was reached. After eliminating the excluded abstracts, we acquired full-text versions of the selected articles, which were rescreened and evaluated more thoroughly for eligibility using the same inclusion and exclusion criteria.

Ultimately, selected studies included (1) adult patients with ROSC after OHCA, (2) those whose BMI was examined, and (3) those who had neurological outcomes recorded at or after discharge and records of in-hospital mortality.

### 2.5. Data Collection Process and Data Items

Two reviewers (H.L. and H.S.) independently extracted the characteristics and results from the selected studies. The following variables were collected from all studies: the name of the first author; year of publication; the country where the study was conducted; inclusion period; study design; study population; number of patients with poor neurological outcome (PNO) and in-hospital mortality; BMI classification; assessment tool of neurological outcome measurement, such as the Cerebral Performance Category (CPC) score; and time points of outcome measurement. Patients were categorized according to the World Health Organization classification as underweight (<18.5 kg/m^2^), normal weight (18.5–24.9 kg/m^2^), overweight (25.0–29.9 kg/m^2^), or obese (≥30 kg/m^2^) [21]. The neurological outcome scores were divided into good or poor based on the CPC score (1–2: good neurological outcome; or 3–5: poor neurological outcome). We requested further information concerning the variables of interest that had not been described in the studies from the corresponding author via email. Studies with insufficient data despite contacting the authors were excluded.

### 2.6. Risk of Bias in Individual Studies

The quality of the included studies was independently estimated by two reviewers (H.L. and H.S.) with blinding to the authors and journal. Risk of bias was estimated using the Risk of Bias Assessment Tool for Non-randomized Studies (RoBANS), with values of 2, 1, and 0 considered as low, unclear, and high risk, respectively [20]. The studies were assessed as having high quality if the sum of the scores for each of the six domains was ≥9 points. A disagreement between the two reviewers was discussed and reviewed by a third author.

### 2.7. Statistical Analysis

In the main analysis, we examined the association between BMI and outcomes in patients with ROSC after OHCA. The strength of the association between BMI and outcomes was calculated as a risk ratio (RR) with a 95% confidence interval (CI) and presented using forest plots. There are two types of statistical models in meta-analysis: random-effects model and fixed effects model. In this study, the random-effects model was used to synthesize the individual data of the included studies, considering variations from the diversity of countries and inclusion periods [22]. Heterogeneity in meta-analysis occurs when observed intervention effects is more different from each other than one would expect due to random errors alone [23]. To measure and quantify heterogeneity, the proportion of between-study inconsistencies using I^2^ tests was estimated, with values of 25%, 50%, and 75% considered to be low, moderate, and high, respectively [24]. Subgroup analysis was performed to investigate the potential causes of heterogeneity based on study quality (high quality study vs. low quality study).

The reference management software, Endnote X9 (Clarivate Analytics, Philadelphia, United States), was used for all identified studies in the literature search. We used Review Manager version 5.3 (Cochrane Collaboration, Oxford, UK) to perform the statistical analysis, and a *p*-value < 0.05 was considered statistically significant.

## 3. Results

### 3.1. Study Selection

**Figure 1:**
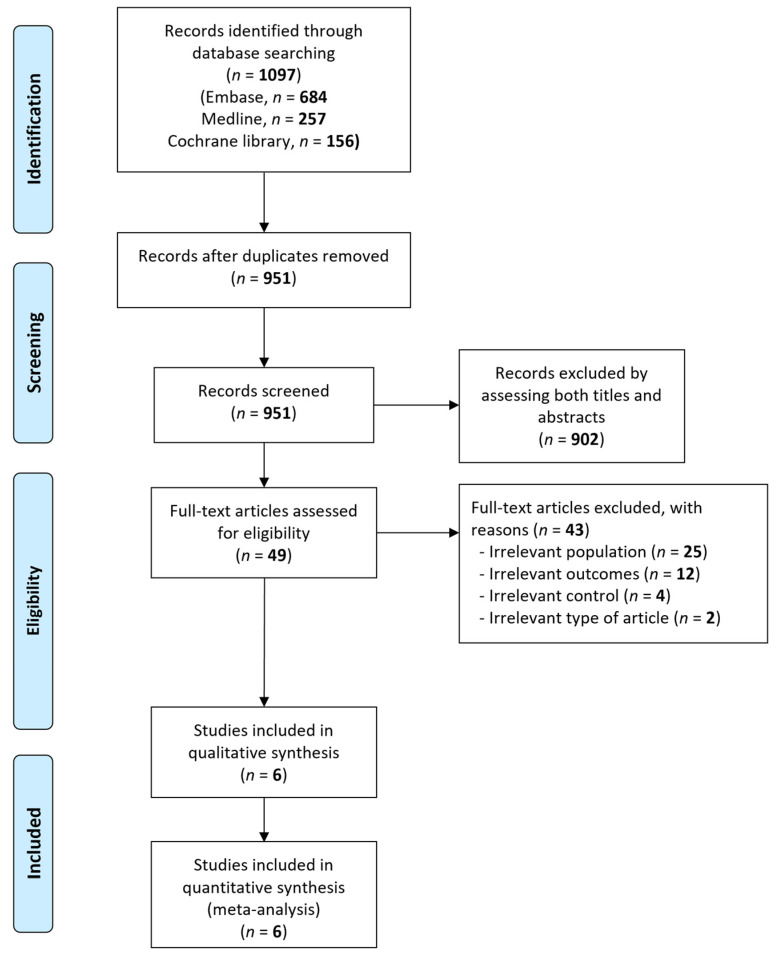
Flow diagram for the identification of relevant studies.

A total of 1097 records were identified from the database search (Figure 1). After excluding 146 duplicates, 951 records were screened for eligibility. Overall, 902 records were excluded after assessing both the title and abstract, and 49 records were identified as being potentially relevant. After reviewing the full-text articles more thoroughly, we excluded 43 studies for irrelevant population (*n* = 25), irrelevant outcome measure (*n* = 12), irrelevant control group (*n* = 4), and meta-analyses (*n* = 2). The six remaining studies with 2427 patients were included in the meta-analysis [13,17,18,25,26,27].

### 3.2. Study Characteristics

The main attributes of all the studies are shown in Table 1, and the characteristics of included patients are presented in Supplementary Table S2. All studies had neurological outcomes as the main outcome measurement, and five studies recorded in-hospital mortality as a patient outcome. The six studies included a total of 2427 patients, with 1639 (67.5%) of those patients having poor neurological outcomes. Of a total of 1910 patients from five studies, 1084 (56.8%) patients had in-hospital mortalities. All the studies assessed neurological outcomes according to the CPC scale. Of the six included studies, five were multicenter, and one was a single-center observational study. The time point of neurological outcome measurement at or after hospital discharge is shown in Table 1.

### 3.3. Risk of Bias in Individual Studies

Of the six studies included in this study, three studies were rated as low-quality studies using the quality scoring system defined in the Methods section [25,26,27]. The major factors that affected study quality were attrition bias by incomplete outcome data and reporting bias. The risk of bias assessment of the studies is summarized in Supplementary Figure S1.

### 3.4. Results of Meta-Analyses

#### 3.4.1. Association between BMI and Neurological Outcome in Patients with ROSC after OHCA

**Figure 2:**
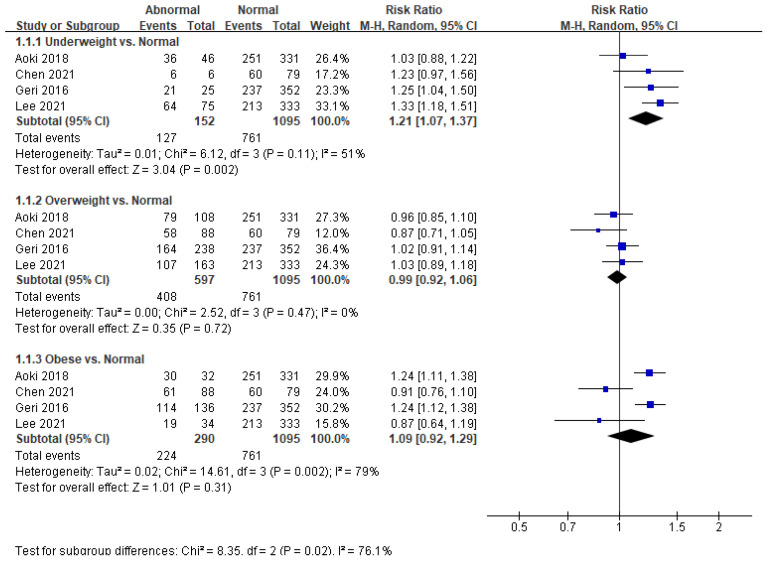
Forest plot of the association between body mass index categories and neurological outcomes in patients with return of spontaneous circulation after out-of-hospital cardiac arrest.

Neurological outcomes were assessed in all the studies (Figure 2). Neurological outcomes in underweight patients were significantly poorer than those in normal-weight patients (RR = 1.21; 95% CI = 1.07–1.37; *p* = 0.002; I^2^ = 51%). There was no significant difference in neurologic outcomes between overweight (*p* = 0.72) or obese (*p* = 0.31) patients and normal-weight patients.

**Figure 3:**
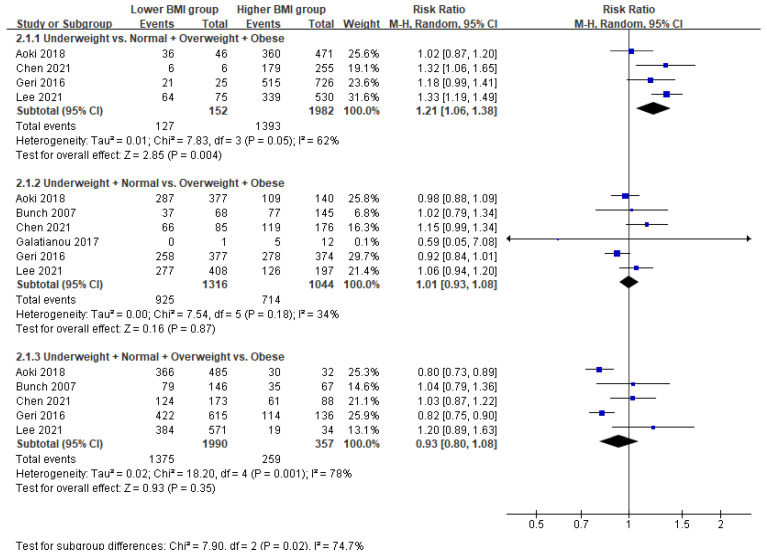
Forest plot of the association between body mass index and neurological outcomes in patients with return of spontaneous circulation after out-of-hospital cardiac arrest.

Additionally, underweight patients had significantly poorer neurological outcomes than did patients who were not underweight (normal-to-obese) (RR = 1.21; 95% CI = 1.06–1.38; *p* = 0.004; I^2^ = 62%) (Figure 3). There was no significant difference between underweight-to-normal weight and overweight-to-obese patients (*p* = 0.87) or between underweight-to-overweight and obese patients (*p* = 0.35). 

Subgroup analysis was performed according to the quality of study (three studies, high quality; one study, low quality) (Supplementary Figure S2). Neurological outcomes in underweight patients were significantly poorer than those in normal-weight patients in the high-quality studies (RR = 1.29; 95% CI = 1.18–1.42; *p* < 0.001; I^2^ = 0%).

#### 3.4.2. Association between BMI and in-Hospital Mortality in Patients with ROSC after OHCA

**Figure 4:**
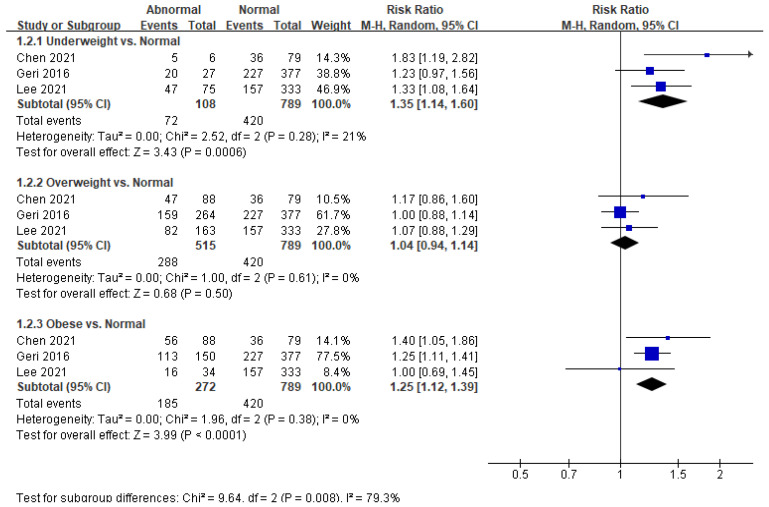
Forest plot of the association between body mass index categories and in-hospital mortality in patients with return of spontaneous circulation after out-of-hospital cardiac arrest.

Five studies reported in-hospital mortality as an outcome (Figure 4). We found that underweight patients showed increased morality (RR = 1.35; 95% CI = 1.14–1.60; *p* < 0.001; I^2^ = 21%) compared with normal-weight patients; similarly, in-hospital mortality in obese patients was poorer than in normal-weight patients (RR = 1.25; 95% CI = 1.12–1.39; *p* < 0.001; I^2^ = 0%). We found no significant difference in in-hospital mortality between overweight and normal-weight patients (*p* = 0.50).

**Figure 5:**
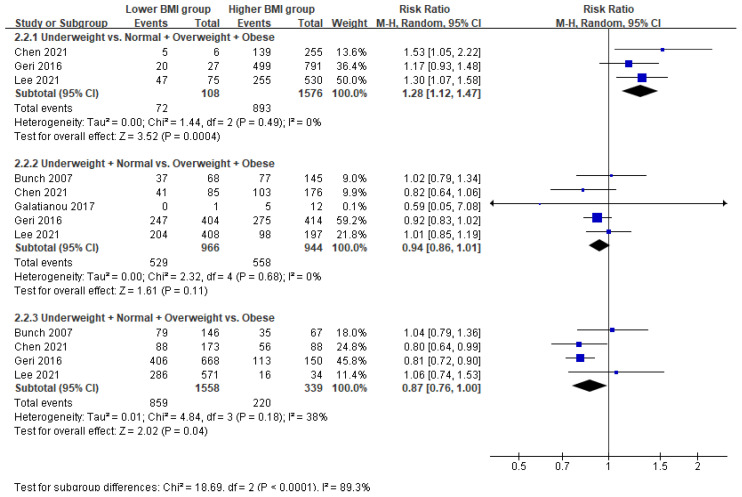
Forest plot of the association between body mass index and in-hospital mortality in patients with return of spontaneous circulation after out-of-hospital cardiac arrest.

Additionally, underweight patients had increased mortality (RR = 1.28; 95% CI = 1.12–1.47; *p* < 0.001; I^2^ = 0%) compared with patients who were not underweight (normal-to-obese). Obese patients had decreased mortality (RR = 0.87; 95% CI = 0.76–1.00; *p* = 0.04; I^2^ = 38%) compared with patients who were not obese (underweight-to-overweight). However, there was no significant difference in mortality between underweight-to-normal weight and overweight-to-obese patients (*p* = 0.11) (Figure 5).

## 4. Discussion

In this study, we aimed to investigate the association between BMI and outcomes in patients with ROSC after OHCA by a meta-analytic approach. We found that underweight patients had significantly poorer neurological outcomes, and underweight and obese patients who could successfully resuscitate from OHCA had higher rates of in-hospital mortality than normal-weight patients. In addition, underweight patients had a lower survival rate than normal-to-obese patients. On the other hand, obese patients had a higher survival rate than underweight-to-overweight patients.

The impact of BMI on outcomes in critically ill patients, including those post cardiac arrest, remains debatable, with previous studies and meta-analyses showing inconsistent results. One recent meta-analysis reported that overweight patients had better survival and neurological prognoses, especially in IHCA, and were managed without TTM [14]. Another meta-analysis showed that being underweight was associated with poor survival outcomes, whereas being overweight was associated with better neurological and survival outcomes, but there were no significant differences when the analysis was restricted to OHCA patients [15]. However, the number of included studies for the subgroup analysis for OHCA patients was relatively small, and one study included both OHCA and IHCA patients [28]. IHCA and OHCA patients have differences in cardiac arrest characteristics, and a recent study reported worse outcomes in OHCA patients than in IHCA patients [3,16]. These limitations may influence the results; therefore, we attempted to improve the quality of analysis for OHCA patients in the present study. In addition, the distribution of BMI categories differs between OHCA and IHCA patients. Therefore, an investigation of a relationship between BMI and outcomes in OHCA patients was needed. 

Several previous studies have reported that a higher BMI had no direct influence on prognosis after cardiac arrest or that obese patients had better outcomes, which is called the obesity paradox [29,30,31]. This phenomenon was observed in post-cardiac-arrest patients in several studies. Testori et al. reported that overweight cardiac arrest patients in Austria had better neurological outcomes in a registry-based, single-center study [29]. Further, one retrospective study in Korea reported that overweight (BMI between 23.0 and 27.5 kg/m^2^) patients treated with TTM had better survival and neurological outcomes than normal-weight patients [28]. Another multicenter, registry-based study that analyzed over 20,000 IHCA patients in the U.S. found that overweight and obese patients had higher survival rates than underweight or normal BMI patients [32]. In contrast, obese patients have a higher risk of comorbidities and OHCA, and anatomical changes in obese patients make high-quality resuscitation, including chest compressions and tracheal intubation, more difficult [27,33,34,35]. Wang et al. reported that a higher BMI and increased thoracic anteroposterior diameter were correlated with poor outcomes in IHCA patients [36]. Geri et al. reported an association between obesity and a high 30-day mortality in OHCA patients treated with TTM [13]. Additionally, no relationship between obesity and worse outcomes was observed in Japanese OHCA patients [25]. In the present study, obese patients showed a higher in-hospital mortality rate than normal-weight patients. This may be influenced by the difficulty of resuscitation and management of post-cardiac-arrest care in obese patients, and these factors are more pronounced in OHCA patients than in IHCA patients. However, obese patients had a lower in-hospital mortality rate than underweight-to-normal patients. Underweight was a poorer prognostic factor than obesity in ROSC patients after OHCA.

Low BMI (underweight patients) is associated with higher all-cause mortality than normal BMI [11,37]. Low BMI is related to poor outcomes in critically ill patients, including patients post cardiac arrest [12]. Malnutrition increases mortality due to impaired wound healing, a higher risk of infection, and complications [38]. Increased morbidity affects the duration of hospitalization and treatment following poor neurological outcomes in patients with chronic or severe disease [38]. Hypocholesterolemia was associated with poor neurological outcomes in post-cardiac-arrest patients in one retrospective observational study [39]. In our present study, underweight patients had poorer neurological outcomes and higher in-hospital mortality than normal BMI patients. Malnutrition and baseline comorbidities, which can cause a patient to be underweight, may increase the risk of poor outcomes. Additionally, underweight cardiac arrest patients less frequently showed cardiogenic arrest with shockable rhythms, which is a good prognostic factor for the etiology of cardiac arrest [13,17].

The analyzed studies in this meta-analysis have heterogeneous baseline characteristics in terms of race, sample size, and proportion of patients treated with TTM. Galatianou et al. included a relatively small number of survivors after cardiac arrest, and the study was conducted at a single center [26]. In one of the included studies, all patients were managed with TTM, and none to one-third of patients underwent TTM in other studies [13]. More effort is demanded to reach and maintain a targeted temperature in obese patients due to larger body mass and lower heat conductivity of fat tissue [40]. Furthermore, patients treated with TTM have poorer initial resuscitation characteristics, such as poorer mental status, than those not treated with TTM. Thus, the heterogeneous proportion of patients treated with TTM in each study may have affected the results of this study. However, based on the results of this study, physicians need to pay more attention when managing underweight or obese OHCA patients, including appropriate airway management and chest compressions for obese patients and nutritional support and neuroprotective treatment for underweight patients.

This study has several limitations. First, as mentioned above, the between-study statistical and clinical heterogeneities were not resolved. This may restrict the quality and interpretation of the findings. Additional analyses, including sensitivity or subgroup analysis and meta-regression, were needed to solve this limitation. However, it was restricted due to the lack of studies. Second, three out of five studies included in the analysis were determined to be of low quality. Well-designed, large-scale prospective studies and meta-analyses are needed to enhance the findings of this study. Third, according to the current guidelines, the recommended timing for neurological outcome measurements is sequentially 1–6 months after cardiac arrest [41]. The included studies in this meta-analysis had a relatively short-term outcome measurement timing, which could have influenced the results of this study. Fourth, the generalizability of our results is limited because the included studies were conducted in developed countries, such as the U.S., Europe, Japan, and Korea. The results of this meta-analysis could be different if included patients were from developing countries with different healthcare systems and socioeconomic/cultural conditions that influence the nutrition of populations. Finally, the nutritional status or preexisting comorbidities that can affect BMI or functional status of patients were not a concern in this study. A large-scale follow-up prospective study with wider representation is needed to enhance the finding of this meta-analysis.

## 5. Conclusions

Obesity was associated with higher in-hospital mortality, whereas underweight was associated with poorer neurological outcome and higher in-hospital mortality compared with normal weight in patients with ROSC after OHCA. A better understanding of the risks associated with obese or underweight patients may lead to better patient outcomes.

## Figures and Tables

**Table 1:** Study characteristics.

Author.	Year	Country	Inclusion Period	Study Type	Sample Size	PNO, *n* (%)	Mortality, *n* (%)	BMI Classification	Time Point of Neurologic Outcome Assessment
Aoki	2018	Japan	2012	mPOS	517	36/46 (78.3) 251/331 (75.8) 79/108 (73.2) 30/32 (93.8)	NR	Underweight (<18.5) Normal (18.5–24.9) Overweight (25.0–29.9) Obese (≥30.0)	1 month
Bunch	2007	USA	1990–2006	mPOS	213	37/68 (54.4) 42/78 (53.9) 35/67 (52.2)	37/68 (54.4) 42/78 (53.9) 35/67 (52.2)	Low to normal weight (<25) Overweight (25–30) Obese (>30)	at hospital discharge
Chen	2021	USA	2011–2018	mROS	261	6/6 (100) 60/79 (75.9) 58/88 (65.9) 61/88 (69.3)	5/6 (83.3) 36/79 (45.6) 47/88 (53.4) 56/88 (63.6)	Underweight (<18.5) Normal (18.5–24.9) Overweight (25.0–29.9) Obese (≥30.0)	at hospital discharge
Galatianou	2017	Greece	2014	sPOS	13	0/1 (0.0) 5/12 (41.7)	0/1 (0.0) 5/12 (41.7)	Normal (<25) Elevated BMI (≥25)	at ICU discharge
Geri	2016	France	2005–2012	mPOS	818	21/25 (84.0) 237/352 (67.3) 164/238 (68.9) 114/136 (83.8)	20/27 (74.1) 227/377 (60.2) 159/264 (60.2) 113/150 (75.3)	Underweight (<18.5) Normal (18.5–25) Overweight (25–30) Obese (>30)	at ICU discharge
Lee	2021	Korea	2015–2018	mPOS	605	64/75 (85.3) 213/333 (64.0) 107/163 (65.6) 19/34 (55.9)	47/75 (62.7) 157/333 (47.1) 82/163 (50.3) 16/34 (47.1)	Underweight (<18.5) Normal (18.5–25) Overweight (25–30) Obese (≥30)	at hospital discharge

Abbreviations, PNO, poor neurologic outcome; BMI, body mass index; TTM, targeted temperature management; mPOS, multi-center prospective observational study; NR, not reported; CPC, cerebral performance categories; sPOS, single-center prospective observational study; ICU, intensive care unit.

## Data Availability

Not applicable.

## Supplementary Materials

The following are available online at https://www.mdpi.com/article/10.3390/ijerph18168389/s1, Table S1: Comprehensive list presenting the search strategy; Table S2: Patients characteristics; Figure S1: Assessment of study quality. Figure S2: Subgroup analysis according to quality of study for the association between body mass index categories and neurological outcomes in patients with return of spontaneous circulation after out-of-hospital cardiac arrest.

## Abbreviations

BMI	body mass index
ROSC	return of spontaneous circulation
OHCA	out-of-hospital cardiac arrest
RR	risk ratio
IHCA	in-hospital cardiac arrest
PNO	poor neurological outcome
CPC	Cerebral Performance Category
CI	confidence interval
TTM	targeted temperature management
