# Supplementary material for: Association between Body Mass Index and Outcomes in Patients with Return of Spontaneous Circulation after Out-of-Hospital Cardiac Arrest: A Systematic Review and Meta-Analysis
Source: Int J Environ Res Public Health. 2021 Aug 8;18(16):8389. doi: 10.3390/ijerph18168389 (PMC8394455; doi:10.3390/ijerph18168389)
Supplement: Supplementary file 1 [file ijerph-18-08389-s001.zip › ijerph-1303221-supplementary.pdf]

Table S1. Comprehensive list presenting the search strategy.

Embase

No.	Searches	Results
1	body mass index.ti,ab.	22667
2	body weight.ti,ab.	13283
3	BMI.ti,ab.	10350
4	obesity.ti,ab.	100038
5	overweight.ti,ab.	22742
6	underweight.ti,ab.	1120
7	1 or 2 or 3 or 4 or 5 or 6	158528
8	(return of spontaneous circulation or ROSC).ti,ab. or exp heart arrest/ or cardiac arrest*.ti,ab. or cardiovascular arrest*.ti,ab. or heart arrest*.ti,ab. or cardiopulmonary arrest*.ti,ab. or asystol*.ti,ab. or pulseless electrical activity.ti,ab. or exp ventricular fibrillation/ or exp advanced cardiac life support/ or (advanced cardiac life support or ACLS).ti,ab. or exp cardiopulmonary resuscitation/ or CPR.mp. or cardiopulmonary resuscitation.ti,ab.	225563
9	7 and 8	684

Medline

No.	Searches	Results
1	body mass index.ti,ab.	15585
2	body weight.ti,ab.	85251
3	BMI.ti,ab.	5398
4	obesity.ti,ab.	70263
5	overweight.ti,ab.	16438
6	underweight.ti,ab.	888
7	1 or 2 or 3 or 4 or 5 or 6	179224
8	(return of spontaneous circulation or ROSC).ti,ab. or exp heart arrest/ or cardiac arrest*.ti,ab. or cardiovascular arrest*.ti,ab. or heart arrest*.ti,ab. or cardiopulmonary arrest*.ti,ab. or asystol*.ti,ab. or pulseless electrical activity.ti,ab. or exp ventricular fibrillation/ or exp advanced cardiac life support/ or (advanced cardiac life support or ACLS).ti,ab. or exp cardiopulmonary resuscitation/ or CPR.mp. or cardiopulmonary resuscitation.ti,ab.	98574
9	7 and 8	257

Cochrane library

No.	Searches	Results
1	MeSH descriptor: [Body Mass Index] explode all trees	10410
2	MeSH descriptor: [Body Weight] explode all trees	29197
3	MeSH descriptor: [Obesity] explode all trees	14556
4	MeSH descriptor: [Overweight] explode all trees	17275
5	MeSH descriptor: [Underweight] explode all trees	297
6	1 or 2 or 3 or 4 or 5	33853
7	MeSH descriptor: [Return of Spontaneous Circulation] explode all trees	1
8	MeSH descriptor: [Heart arrest] explode all trees	2001
9	MeSH descriptor: [Resuscitation] explode all trees	5207
	MeSH descriptor: [Advanced Cardiac Life Support] explode all trees	60
	#7 or #8 or #9 or #10	6496
15	#6 and #11	156

Table S2. Patients characteristics.

Author	N	BMI, kg/m²	Age, year	Male sex, %	Witnessed, %	Bystander CPR, %	Shockable rhythm, %	HTN, %	DM, %	TTM, %
Aoki	517	Total	-	-	-	-	-	-	-	33.8
Bunch	68	< 25	67.3 ± 14.2	73	75	59	1.8	31	12	0
	78	25-30	62 ± 13.3	81	82	41	1.7	34	16	0
	67	> 30	61.0 ± 12.5	81	92	46	1.8	38	30	0
Chen	6	<18.5	55.5 ± 27.2	50	33.3	0	0	16.7	0	100
	79	18.5-25	54.0 ± 16.6	58.2	45.6	49.4	43.0	40.5	21.5	100
	88	25-30	59.3 ± 17.9	71.6	48.9	36.4	45.5	45.5	14.8	100
	88	>30	59.6 ± 15.2	53.4	68.2	37.5	44.3	58.0	43.2	100
Galatianou	16	< 25	58.8 ± 12.3	68.75	-	-	6.3	-	-	0
	68	> 25	59.4 ± 12.6	73.5	-	-	16.2	-	-	0
Geri	818	Total	60.9 (50.8-72.7)	70.2	88.8	52.6	54.3	44.5	18.3	100
	27	<18.5	58.0 (45.0-77.2)	44.4	81.5	18.5	33.3	18.5	0	100
	377	18.5-25	58.7 (47.7-72.7)	68.2	84.9	40.6	53.6	23.9	9.8	100
	264	25-30	60.9 (51.1-72.4)	79.9	89.4	46.2	59.1	36.4	12.5	100
	150	>30	65.1 (55.9-72.6)	62.7	91.3	43.3	51.3	47.3	24.7	100
Lee	605	Total	63 (53–74)	69.75	74.21	51.0	41.88	48.76	31.51	37.19
	75	<18.5	71 (62–79)	70.67	73.33	52.7	14.86	41.33	21.33	22.67
	333	18.5-24.9	63 (54–75)	69.97	73.27	49.4	45.29	46.08	30.72	36.64
	163	25-29.9	61 (51–72)	70.55	76.69	51.5	45.00	57.41	37.04	44.17
	34	≥30	56.5 (42–72)	61.76	73.53	60.6	52.94	50	35.29	41.18

Figure S1. Assessment of study quality.

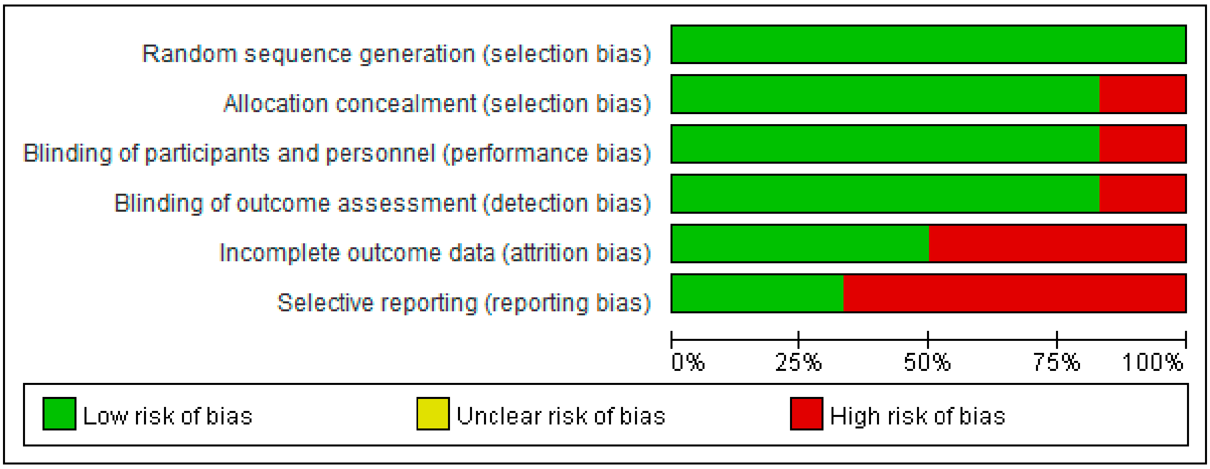

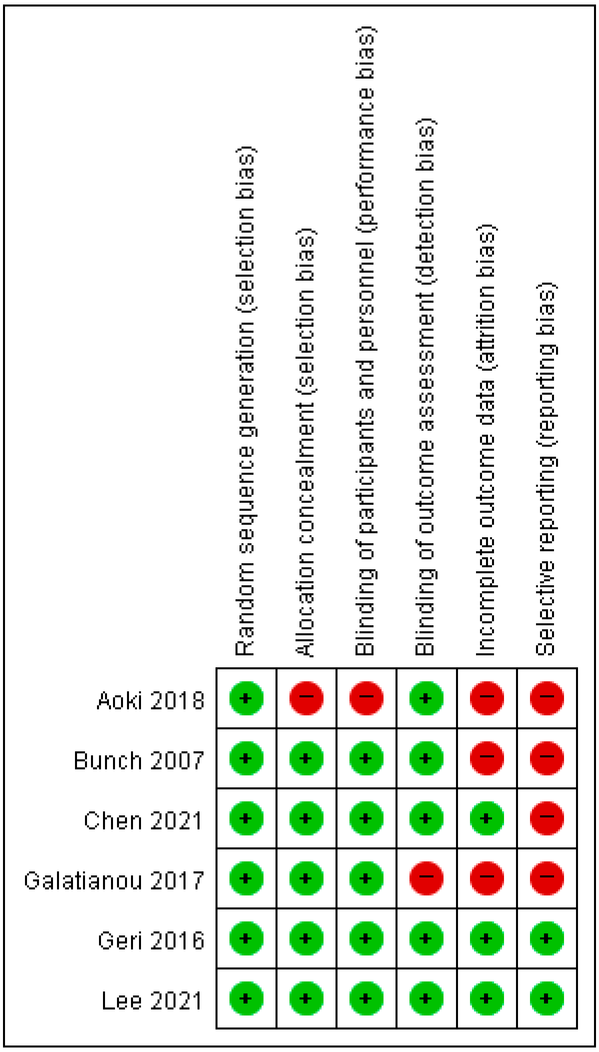

Figure S2. Subgroup analysis according to quality of study for the association between body mass index categories and neurological outcomes in patients with return of spontaneous circulation after out-of-hospital cardiac arrest.

	Abnormal		Normal			Risk Ratio
Study or Subgroup	Events	Total	Events	Total	Weight	M-H, Random, 95% CI
**3.1.1 Underweight vs. Normal (High quality study)**						
Chen 2021	6	6	60	79	15.5%	1.23 [0.97, 1.56]
Geri 2016	21	25	237	352	25.9%	1.25 [1.04, 1.50]
Lee 2021	64	75	213	333	58.5%	1.33 [1.18, 1.51]
**Subtotal (95% CI)**		**106**		**764**	**100.0%**	**1.29 [1.18, 1.42]**
Total events	91		510			
Heterogeneity: Tau² = 0.00; Chi² = 0.58, df = 2 (P = 0.75); I² = 0%						
Test for overall effect: Z = 5.34 (P < 0.00001)						
**3.1.2 Underweight vs. Normal (Low quality study)**						
Aoki 2018	36	46	251	331	100.0%	1.03 [0.88, 1.22]
**Subtotal (95% CI)**		**46**		**331**	**100.0%**	**1.03 [0.88, 1.22]**
Total events	36		251			
Heterogeneity: Not applicable						
Test for overall effect: Z = 0.38 (P = 0.71)						
**3.1.3 Overweight vs. Normal (High quality study)**						
Chen 2021	58	88	60	79	18.4%	0.87 [0.71, 1.05]
Geri 2016	164	238	237	352	47.5%	1.02 [0.91, 1.14]
Lee 2021	107	163	213	333	34.1%	1.03 [0.89, 1.18]
**Subtotal (95% CI)**		**489**		**764**	**100.0%**	**0.99 [0.91, 1.08]**
Total events	329		510			
Heterogeneity: Tau² = 0.00; Chi² = 2.33, df = 2 (P = 0.31); I² = 14%						
Test for overall effect: Z = 0.14 (P = 0.89)						
**3.1.4 Overweight vs. Normal (Low quality study)**						
Aoki 2018	79	108	251	331	100.0%	0.96 [0.85, 1.10]
**Subtotal (95% CI)**		**108**		**331**	**100.0%**	**0.96 [0.85, 1.10]**
Total events	79		251			
Heterogeneity: Not applicable						
Test for overall effect: Z = 0.55 (P = 0.59)						
**3.1.5 Obese vs. Normal (High quality study)**						
Chen 2021	61	88	60	79	34.5%	0.91 [0.76, 1.10]
Geri 2016	114	136	237	352	39.2%	1.24 [1.12, 1.38]
Lee 2021	19	34	213	333	26.3%	0.87 [0.64, 1.19]
**Subtotal (95% CI)**		**258**		**764**	**100.0%**	**1.02 [0.79, 1.32]**
Total events	194		510			
Heterogeneity: Tau² = 0.04; Chi² = 11.67, df = 2 (P = 0.003); I² = 83%						
Test for overall effect: Z = 0.14 (P = 0.89)						
**3.1.6 Obese vs. Normal (Low quality study)**						
Aoki 2018	30	32	251	331	100.0%	1.24 [1.11, 1.38]
**Subtotal (95% CI)**		**32**		**331**	**100.0%**	**1.24 [1.11, 1.38]**
Total events	30		251			
Heterogeneity: Not applicable						
Test for overall effect: Z = 3.84 (P = 0.0001)						

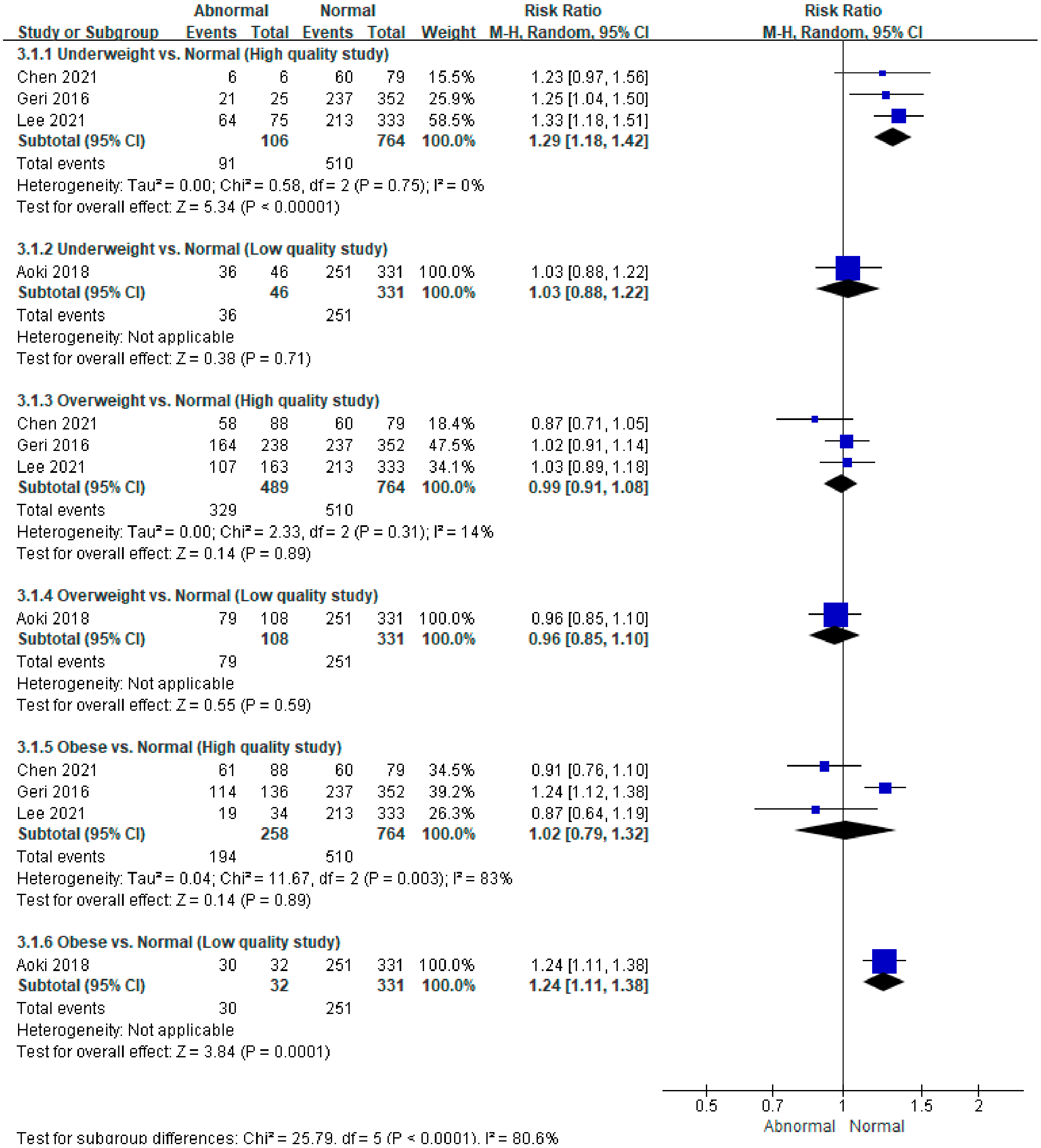

Test for subgroup differences: Chi² = 25.79, df = 5 (P < 0.0001), I² = 80.6%
